# Immunomodulatory Effects and Protection in Sepsis by the Antibiotic Moxifloxacin

**DOI:** 10.3390/antibiotics13080742

**Published:** 2024-08-07

**Authors:** Tiago R. Velho, Helena Raquel, Nuno Figueiredo, Ana Neves-Costa, Dora Pedroso, Isa Santos, Katharina Willmann, Luís F. Moita

**Affiliations:** 1 Department of Cardiothoracic Surgery, Hospital de Santa Maria, Unidade Local de Saúde de Santa Maria, Av. Prof. Egas Moniz, 1649-035 Lisbon, Portugal; tvelho@igc.gulbenkian.pt; 2 Cardiothoracic Surgery Research Unit, Centro Cardiovascular da Universidade de Lisboa (CCUL@RISE), Faculdade de Medicina da Universidade de Lisboa, 1649-028 Lisbon, Portugal; 3 Innate Immunity and Inflammation Laboratory, Instituto Gulbenkian de Ciência, 2780-156 Oeiras, Portugalnunolfigueiredo@hotmail.com (N.F.); ananevescostaana@gmail.com (A.N.-C.); dcpedroso@igc.gulbenkian.pt (D.P.);; 4 Department of General Surgery, Hospital Lusíadas Lisboa, 1500-458 Lisbon, Portugal; 5 Department of General Surgery, Hospital de São Bernardo, Unidade Local de Saúde da Arrábida, 2910-446 Setúbal, Portugal; 6 Center for Disease Mechanisms Research, Faculdade de Medicina da Universidade de Lisboa, 1649-028 Lisbon, Portugal

**Keywords:** IL-1β, TNF-α, sepsis, moxifloxacin, quinolones, antibiotics

## Abstract

Sepsis is a leading cause of death in Intensive Care Units. Despite its prevalence, sepsis remains insufficiently understood, with no substantial qualitative improvements in its treatment in the past decades. Immunomodulatory agents may hold promise, given the significance of TNF-α and IL-1β as sepsis mediators. This study examines the immunomodulatory effects of moxifloxacin, a fluoroquinolone utilized in clinical practice. THP1 cells were treated in vitro with either PBS or moxifloxacin and subsequently challenged with lipopolysaccharide (LPS) or *E. coli*. C57BL/6 mice received intraperitoneal injections of LPS or underwent cecal ligation and puncture (CLP), followed by treatment with PBS, moxifloxacin, meropenem or epirubicin. Atm^−/−^ mice underwent CLP and were treated with either PBS or moxifloxacin. Cytokine and organ lesion markers were quantified via ELISA, colony-forming units were assessed from mouse blood samples, and DNA damage was evaluated using a comet assay. Moxifloxacin inhibits the secretion of TNF-α and IL-1β in THP1 cells stimulated with LPS or *E. coli*. Intraperitoneal administration of moxifloxacin significantly increased the survival rate of mice with severe sepsis by 80% (*p* < 0.001), significantly reducing the plasma levels of cytokines and organ lesion markers. Notably, moxifloxacin exhibited no DNA damage in the comet assay, and Atm^−/−^ mice were similarly protected following CLP, boasting an overall survival rate of 60% compared to their PBS-treated counterparts (*p* = 0.003). Moxifloxacin is an immunomodulatory agent, reducing TNF-α and IL-1β levels in immune cells stimulated with LPS and *E. coli*. Furthermore, moxifloxacin is also protective in an animal model of sepsis, leading to a significant reduction in cytokines and organ lesion markers. These effects appear unrelated to its antimicrobial activity or induction of DNA damage.

## 1. Introduction

Sepsis is a complex syndrome that results from a harmful and damaging dysregulated systemic inflammatory response to infection [1], leading to organ dysfunction and a heightened risk of mortality [2]. Its overall mortality rate is approximately 30%, rising to 40% in the elderly and nearly 50% in patients experiencing septic shock [3,4]. While the precise incidence of sepsis varies by country, sepsis stands as the leading cause of death among critically ill patients. In 2017 alone, it accounted for an estimated 48.9 million cases recorded and 11 million sepsis-related deaths globally, representing 19.7% of all recorded deaths [5]. Moreover, this pervasive health challenge carries a substantial economic burden [6]. 

Sepsis arises from both community-acquired and healthcare-associated infections caused by a wide range of infecting organisms [7,8]. The lung is the primary site of infection in over half of these cases, followed by intra-abdominal, bloodstream, renal, and urinary tract infections. Currently, Gram-positive bacteria may be more prevalent than Gram-negative strains, although certain studies suggest a comparable occurrence [8]. 

Various infectious triggers activate numerous immune cells, including macrophages, monocytes and neutrophils [9], leading to the production of multiple cytokines, such as interleukin 1β (IL-1β) and tumor necrosis factor α (TNF-α) [10]. TNF-α and IL-1β serve as prominent inflammatory mediators initiating the immunopathological features of sepsis-induced shock. They trigger a secondary cascade of inflammatory responses involving cytokines, lipid mediators and reactive oxygen species. Additionally, they upregulate cell adhesion molecules and inflammatory cell migration into tissues [11].

There is no effective specific therapy to treat sepsis, with the current approach primarily focused on supportive organ measures and controlling the primary infection source. Presently, prompt antibiotic therapy stands as the cornerstone of sepsis treatment, alongside fluid resuscitation and, if required, vasopressors. For every hour without appropriate antibiotic coverage from the onset of signs and symptoms of sepsis, mortality increases by 4% [12]. Despite considerable efforts in the field, significant advances in sepsis treatment have remained stagnant over the past four decades.

Based on the importance of TNF-α and IL-1β as inflammatory cytokines in sepsis, our laboratory conducted drug screening involving over 2300 compounds to identify those capable of simultaneously inhibiting TNF-α and IL-1β secretion [13]. Notably, among the most potent inhibitors of both cytokines, we found moxifloxacin (MFX), a fluoroquinolone antibiotic. 

Our study aimed to explore whether MFX administration could serve a dual purpose in sepsis management: exhibiting antimicrobial activity while modulating the beneficial immune host response. For this, we employed both an in vitro cell line and a sepsis mouse model. Furthermore, our goal was to elucidate the molecular mechanisms underlying its protective effect.

## 2. Results

### 2.1. Moxifloxacin Inhibits the Secretion of IL-1β and TNF-α 

To determine the immunomodulatory effects of MFX, THP-1 cells were stimulated with *E. coli* or LPS in the presence or absence of MFX at different concentrations (5, 10, or 20 μM). Cells were incubated with the stimuli for 4 h, 6 h, 8 h, 16 h and 24 h. Exposure of THP-1 cells to *E. coli* and LPS for a short period (6 h) induced a marked increase in the secretion of TNF-α by the cells, but a longer period (16 h) was necessary to induce an increase in the secretion of IL-1β. 

**Figure 1:**
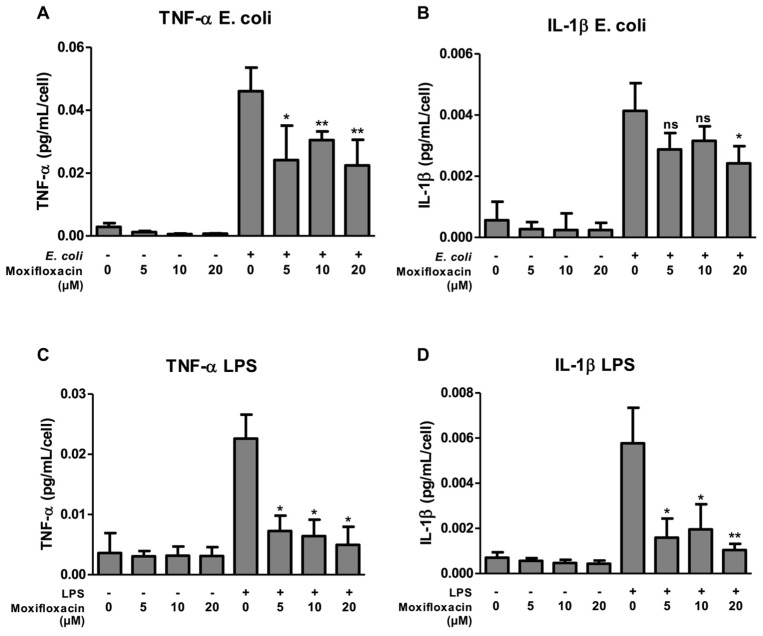
Moxifloxacin inhibits the secretion of IL-1β and TNF-α. THP-1 cells were incubated with *Escherichia coli* (**A**,**B**) or lipopolysaccharide (LPS) (**C**,**D**), with moxifloxacin at concentrations of 5, 10 and 20 μM. IL-1β and TNF-α were reduced with both the stimuli. ns: non-significant; *: *p* < 0.05; **: *p* < 0.01.

Exposure of *E. coli*-stimulated THP-1 cells to MXF at concentrations of 5, 10 and 20 μM resulted in statistically significant reductions in the secretion of TNF-α by 47.6% (*p* < 0.05), 33.8% and 51.2% (*p* < 0.01), respectively (Figure 1A). The secretion of IL-1β was also reduced with the three concentrations used, by 30.5% (ns), 23.6% (ns) and 41.4% (*p* < 0.05), respectively. However, only with the 20 μM concentration was a statistically significant reduction achieved (Figure 1B). 

Similarly, the stimulation of THP-1 cells with LPS increased the secretion of IL-1β and TNF-α as expected. MXF inhibited the induction of TNF-α in LPS-stimulated cells by 67.9%, 71.6% and 78% at concentrations of 5, 10 and 20 μM, respectively (*p* < 0.05) (Figure 1C). The same inhibition was observed in the secretion of IL-1β (reduction of 72.4%, 66.1% and 81.9% at concentrations of 5 (*p* < 0.05), 10 (*p* < 0.05) and 20 μM (*p* < 0.01), respectively) [Figure 1D].

Cells were not previously activated, since non-stimulated cells had no cytokine production (Figure 1A–D). Moreover, toxicity of MXF for the cultured THP-1 was not observed at any concentrations used, as determined by an Alamar Blue test.

### 2.2. Moxifloxacin Protects against Severe Sepsis

**Figure 2:**
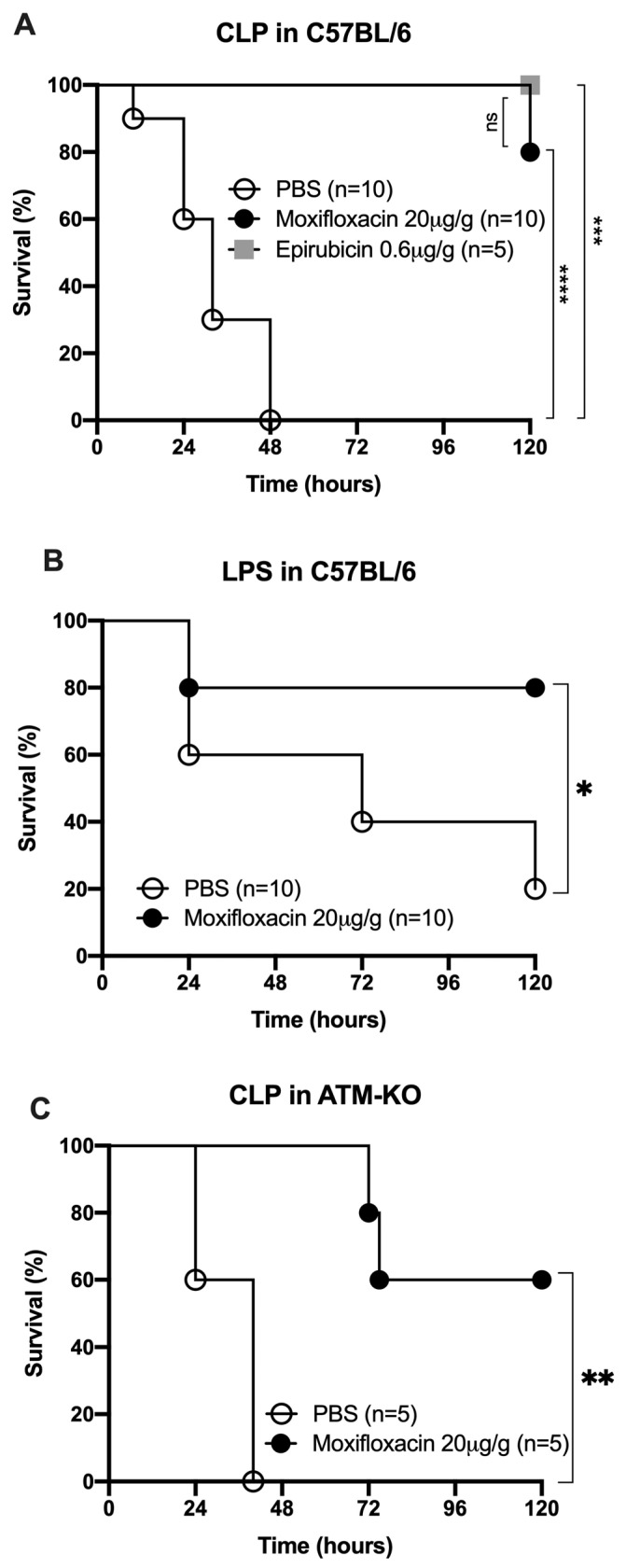
Moxifloxacin protection against sepsis. (**A**) Survival of C57BL/6 WT mice subjected to CLP treated with carrier (PBS), epirubicin (0.6 μg/g body weight) or moxifloxacin (20 μg/g body weight), at time of procedure and 24 h later. (**B**) Survival of C57BL/6 WT with LPS treated with PBS or moxifloxacin (20 μg/g body weight). (**C**) Survival of ATM-KO mice subjected to CLP treated with carrier (PBS) or moxifloxacin (20 μg/g body weight) at time of procedure and 24 h later. ns: non-significant; *: *p* < 0.05; **: *p* < 0.01; ***: *p* < 0.001; ****: *p* < 0.0001.

To investigate the in vivo effects of MFX, we used the cecal ligation and puncture (CLP) mouse model. Polymicrobial sepsis of abdominal origin induced by CLP is a commonly used model of sepsis, simulating many of the circulatory and metabolic alterations produced by sepsis [14,15]. The CLP model is currently considered the gold standard in sepsis research, with better simulation of the circulatory and metabolic alterations produced by sepsis than endotoxin animal models [16,17]. We adjusted the CLP severity to high-grade sepsis, where all the C57BL/6 mice died within the first 48 h after the procedure. MFX, administered intraperitoneally (20 μg/g of body weight) right after the CLP and 24 h later, increased the survival of mice with severe sepsis by 80% (*p* < 0.001) (Figure 2A). We used, as a positive control, treatment with epirubicin (0.6 μg/g of body weight), as epirubicin confers protection against severe sepsis [13].

**Figure 3:**
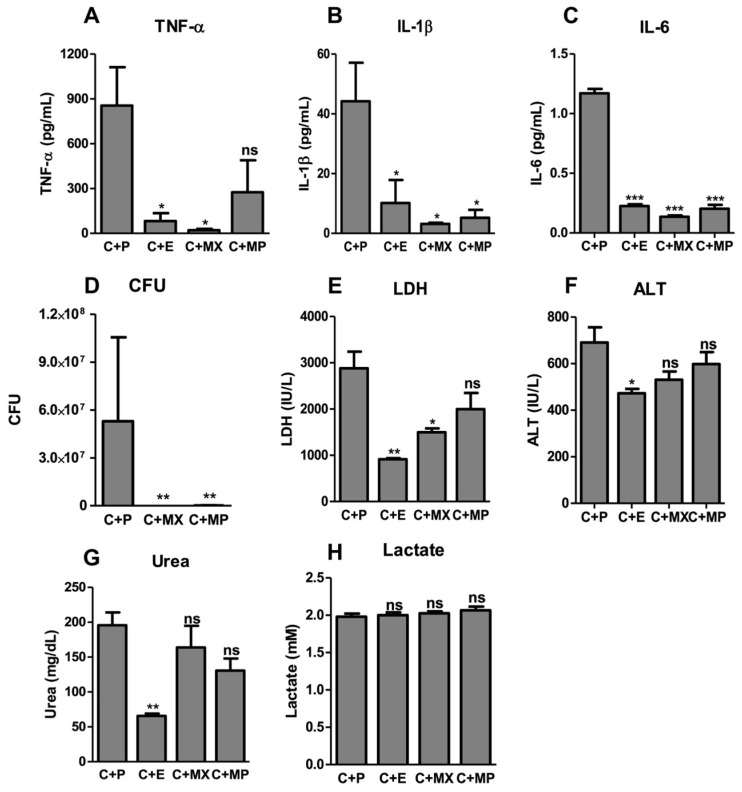
Moxifloxacin has anti-inflammatory and protective effects in vivo. Moxifloxacin reduces inflammation and tissue lesions associated with CLP, as assessed by the reduced plasma levels of TNFα (**A**), IL1β (**B**), IL6 (**C**), and LDH (**E**), ALT (**F**) and urea (**G**) in C57BL/6 WT animals 24 h after CLP followed by treatment with PBS (C + P), epirubicin (C + E), moxifloxacin (C + MX) or meropenem (C + MP). Lactate levels were not changed between all groups (**H**). Polymicrobial load (CFU) in blood (**D**) of C57BL/6 WT animals 24 h after CLP followed by treatment with PBS (C + P), moxifloxacin (C + MX) or meropenem (C + MP). ns: non-significant; *: *p* < 0.05; **: *p* < 0.01; ***: *p* < 0.001.

We also tested the efficiency of MFX in comparison to meropenem (40 μg/g of body weight), a large-spectrum antibiotic widely used in sepsis [2]. Both MXF and meropenem effectively reduced bacterial load in CLP-subjected mice compared to non-treated mice (Figure 3D). However, only MFX protected mice against sepsis, as meropenem could only delay the kinetics of death in sepsis [13]. This difference in the protective role in sepsis between these two antibiotics argues in favor of effects beyond the direct antibiotic effect of MFX. 

Protection against severe sepsis was confirmed with the observation that TNF-α (Figure 3A), IL-1β (Figure 3B), IL-6 (Figure 3C), LDH (Figure 3E), ALT (Figure 3F) and urea (Figure 3G) levels were decreased to almost basal levels in MFX-treated mice 24 h after CLP compared to untreated mice. Interestingly, all tested groups had similar lactate levels (Figure 3H). Although lactic acid is a good marker of hypoperfusion and cellular derangements in humans, as well as a good predictor of multiple system organ failure and death [2], this may not be true in mice, since it has been shown that lactic acid remains unchanged in intestinal ischemia and CLP mice models [18]. 

### 2.3. Moxifloxacin Action Is Not Due to DNA Damage 

MFX inhibits homologous type II topoisomerase, DNA gyrase and DNA topoisomerase IV, which are essential for controlling DNA topology, chromosome function, and replication [19]. The binding of fluoroquinolones to the DNA–topoisomerase complexes stabilizes the “cleavable complex”, a mechanism similar to that observed for several topoisomerase II inhibitors, such as etoposide [20]. Moreover, high concentrations of some fluoroquinolones have been reported to exhibit genotoxic effects in eukaryotic systems through topoisomerase inhibition. This includes inducing topoisomerase II-mediated DNA cleavage (by enhancing pre- and post-strand DNA breaks) or inhibiting catalytic DNA strand passage activity [19,20,21]. 

**Figure 4:**
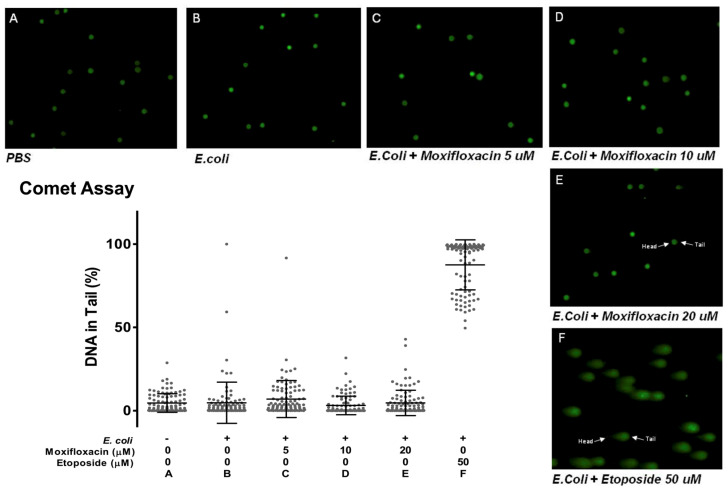
DNA damage in THP-1 cells treated with moxifloxacin and etoposide. Comet assay performed on THP-1 cells verified that DNA damage is similar in all groups incubated with PBS (**A**), *E. coli* (**B**), and with moxifloxacin (4 h), at 5 (**C**), 10 (**D**) and 20 (**E**) μM following stimulation with *E. coli*. On the other hand, treatment with etoposide (**F**) resulted in increased average of DNA in tail, representing high DNA damage.

As MFX (at a concentration of 20 or 40 μg/mL) inhibits human topoisomerase II activity [19], we decided to test the hypothesis that in vitro immunomodulatory effects could be achieved by causing DNA damage to the THP-1 cells. We performed single-cell gel electrophoresis (comet assay) [Figure 4] using etoposide as a positive control. Etoposide is a topoisomerase II inhibitor that can generate DNA double-strand breaks. It acts not only during replication, but also during transcription, therefore inducing DNA damage even in non-replicating cells [22]. 

The comet assay can detect double-stranded breaks, apyrimidinic/apurinic (AP) sites and single-stranded breaks when using an alkali treatment [23]. Electrophoresis causes migration of the broken DNA, which migrates faster than intact DNA. 

Indeed, etoposide revealed long comet tails, indicative of DNA breaks, with mean DNA in the tail of 87.5% (SD ± 15.01). As a control, we analyzed data from THP-1 alone or stimulated with *E. coli*: these cells showed vestigial comet tails (the DNA remained confined within the nuclear environment), with average DNA in the tail of 6.41% (SD ± 14.57) and 4.75% (SD ± 12.34), respectively. MXF showed no apparent DNA damage, as treated cells in concentrations of 5, 10 and 20 μM had comet tails that were close to absent, with mean DNA in the tail of 6.97% (SD ± 11.13), 3.14% (SD ± 5.56) and 4.65% (SD ± 7.61) [Figure 4B]. 

To test whether DNA damage was important in the in vivo action of MFX, we tested the effect of the drug in ataxia telangiectasia-mutated (ATM)-deficient mice. ATM plays an essential role in the maintenance of genome stability, being central to the DNA damage response to DNA double-strand breaks [24].

ATM-deficient (Atm^−/−^) mice were also protected by MFX following CLP, with an overall survival of 60%, compared to 0% of the PBS-treated mice (*p* = 0.003) (Figure 2B). We concluded that ATM expression is not necessary for the protective role of MFX.

## 3. Discussion

Here, we show that the immunomodulatory effects of MFX protect against severe sepsis in mice. When administered after the onset of infection and again 24 h later, MFX effectively reduces cytokine levels—including IL-1β, TNF-α and IL-6—as well as organ lesion markers, such as LDH, ALT and urea.

Fluoroquinolones, renowned for their potent antibacterial activity and broad spectrum, have been a mainstay in clinical practice since the 1980s. MXF, a fourth-generation synthetic methoxyfluoroquinolone, has demonstrated efficacy across various clinical indications over its 25-year usage history. Notably, MXF is recommended as a first-line therapy in patients with nosocomial pneumonia with suspected low risk of resistance and early-onset infection [25]. Additionally, it is utilized in combination therapy to reduce mortality and adverse clinical outcomes in severe community-acquired pneumonia. It has been suggested that supratherapeutic doses of fluoroquinolones exert immunomodulatory effects by inhibiting mammalian topoisomerase type II enzymes [19,20]. While various antibiotic classes, including macrolides, lincosamides, tetracyclines and fluoroquinolones, have been associated with immunomodulation [21,26], recent studies underscore MFX’s impact on cytokine secretion in vitro [27,28,29] and its potential to confer protection against septic shock induced-by LPS [30]. Nevertheless, despite these clinical implications and emerging evidence, investigations into the immunomodulatory effects of MFX in sepsis remains limited. 

Recent reports suggest that combined antimicrobial therapy might be beneficial in treating sepsis, owing to various mechanisms, including the potential beneficial immunomodulatory effects of the secondary agent [26,31,32]. Several classes of antibiotics may have effects beyond their antibacterial properties, as demonstrated recently for tetracyclines [33]. Tetracyclines offer protection against sepsis by activating disease tolerance mechanisms through disruption of the mitochondrial electron transport chain, beyond their direct antibacterial activity. They also reduce lung tissue damage, enhance fatty acid oxidation, and improve glucocorticoid sensitivity in the liver [33]. Similarly, macrolides, another class of antibiotics, exhibit anti-inflammatory effects, with strong evidence supporting their role in reducing exacerbations in patients with chronic obstructive pulmonary disease, asthma, bronchiectasis and cystic fibrosis [34]. Moreover, macrolides appear to provide clinical benefits and protection in patients infected with macrolide-tolerant organisms, such as *Pseudomonas aeruginosa* [34].

Indeed, a randomized trial suggested that adding MFX to meropenem would leverage different synergistic mechanisms in treating sepsis. However, it did not lead to improved outcomes in the studied population, as there were no significant differences in the occurrence of organ failure between patients receiving meropenem alone compared to those receiving meropenem combined with MFX [35]. It is important to highlight that in this trial, MFX was administered intravenously at a dosage of 400 mg per day for seven days, while in our study, we administered MFX twice at a dose equivalent to 1000 mg in humans. In our study, we used MFX at 20 µg/g body weight and meropenem at 40 µg/g body weight, which is equivalent to 1000 mg and 2000 mg, respectively, for a human adult weighing 50 kg. It is plausible that the immunomodulatory effects of MFX may only be achieved with higher doses.

The precise mechanism underlying the proposed immunomodulatory effects of fluoroquinolones remains unknown, although it has been suggested that these compounds can inhibit mammalian topoisomerase type II enzymes [19,20]. Studies have demonstrated that low levels of DNA damage to the host, as observed with the administration of anthracyclines, induce protection in sepsis by activating disease tolerance mechanisms [13]. However, in our study, the immunomodulatory effects do not appear to be linked to DNA damage, as no DNA damage was detected upon MFX administration. Furthermore, ATM-deficient mice, which lack the *ATM* gene essential in the DNA damage response, showed similar protection to wild-type mice upon MFX administration, as previously discussed. 

The precise mechanism underlying the immunomodulatory effects of MFX remains elusive, although insights from the immune effects of other quinolones may establish a background for further research endeavors. For instance, ciprofloxacin has been noted to improve innate-immunity-mediated pregnancy outcomes by stimulating the production of granulocyte–macrophage colony-stimulating factor (GM-CSF) [36]. This trend of GM-CSF production was similarly observed in mice with experimental antiphospholipid syndrome following treatment with ciprofloxacin [37]. 

## 4. Materials and Methods

### 4.1. Compounds

Moxifloxacin (MXF), meropenem, etoposide, epirubicin and lipopolysaccharide (LPS) were purchased from Sigma (Missouri, IL, USA).

### 4.2. Cell Culture

THP-1 cells (monocyte/macrophage cell line—American Tissue Culture Collection—ATCC TIB-202) were cultured in RPMI medium supplemented with 10% (*v*/*v*) Fetal Bovine Serum, 1% (*v*/*v*) penicillin–streptomycin, 1% (*v*/*v*) pyruvate, 1% (*v*/*v*) L-glutamine, 1% (*v*/*v*) non-essential amino acids, 1% (*v*/*v*) Hepes buffer and 0.05 M 2-Mercaptoethanol. The cells were kept at 37 °C under a 5% carbon dioxide (CO_2_) atmosphere.

### 4.3. IL-1β and TNF-α Secretion

THP-1 cells were plated in 96-well plates at 5 × 10^6^ cell/mL, and then, they were stimulated with LPS or 4% PFA-fixed DH5 *Escherichia coli* (*E. coli*) at a Multiplicity of Infection (MOI) of 20 bacteria cells per THP-1 cell, 1 h after incubation with MFX at 5 µM, 10 µM or 20 µM. After 4 h, 6 h, 8 h, 16 h and 24 h periods, cell viability was assessed by an Alamar Blue test (Invitrogen, Waltham, MA, USA), according to the manufacturer’s instructions, and the cell supernatants were collected. IL-1β and TNF-α cytokines were quantified by an enzyme-linked immunosorbent assay (ELISA) using Human IL-1β ELISAMAX and Human TNF-α ELISAMAX (BioLegend, San Diego, CA, USA), respectively, according to the company’s protocol. All data values from IL-1β and TNF-α secretion assays were normalized by dividing the amount of IL-1β and TNF-α in the conditioned medium 4 h, 6 h, 8 h, 12 h or 24 h after *E. coli* stimulation by the number of cells in each well and then by the average concentration per cell of the plate.

### 4.4. Comet Assay (Single-Cell Gel Electrophoresis)

This assay was used for the quantification of DNA damage. The comet assay was performed using the CometAssay kit following the manufacturer’s (Trevigen, Gaithersburg, MD, USA) instructions. Briefly, an aliquot of 5 µL of cells (1 × 10^5^ cell/mL) was added to 50 µL of molten LMAgarose (0.5% low-melting agarose) kept at 37 °C. After mixing the sample, a 55 µL aliquot was pipette onto an area of the CometSlide. The slide was incubated at 4 °C in the dark for 10 min to accelerate gelling of the agarose disk and then transferred to prechilled Lysis Solution for 30 min at 4 °C. A denaturation step was performed in alkali solution at room temperature for 30 min, shielded from light. The slide was then transferred to horizontal electrophoresis apparatus (1 Volt/cm for 40 min; buffer was adjusted to 300 mA current). The slide was then immersed in 70% ethanol for 5 min and air dried for 15 min. For observation, samples were stained with SYBR^®^ Green (Molecular Probes, Eugene, OR, USA), diluted 1:10,000 in 10 mM Tris-Cl pH 7.5 and 1 mM EDTA and observed by epifluorescence microscopy with a 494 nm filter. The results were analyzed using CometScore^®^ 2.0 software. The parameters for analysis consisted of measuring the length of the comet tail and estimating the distribution of the fluorescence between the head of the comet and the tail.

### 4.5. Animal Experimental Design

All the procedures were conducted in accordance with the Portuguese guidelines and regulations after approval by the respective local committee (Instituto Gulbenkian de Ciência). All mice used were 8–12 weeks old. The mice were bred and maintained under specific pathogen-free (SPF) conditions. The C57BL/6 mice were obtained from Charles River laboratories. On pre-warmed heat pads, animals were anesthetized using a xylazine/ketamine mixture (0.8 mL 2% xylazine; 1.2 mL ketamine; 8 mL saline—10 µL/g body weight) or isoflurane. The abdomen was disinfected with chlorhexidine scrub and solution, betadine and alcohol. To expose the cecum, a longitudinal para-midline incision was made, ensuring that the peritoneal cavity was not penetrated. The cecum was located and pulled out. Feces were pushed to the tip of the cecum. The cecum was ligated at 50% of the distance between the distal pole and the base of the cecum. The extent of ligation determined the severity of the sepsis [14,15]. The cecum was perforated by a single “through and through” puncture midway to the ligation and the tip of the cecum with a needle in a mesenteric-to-antimesenteric direction. The cecum was then relocated into the abdominal cavity without spreading feces on the abdominal wall or wound margins. The peritoneum and abdominal muscles were closed with running sutures, and the skin was closed using metallic wound clips. The animals were rehydrated by injecting prewarmed saline (37 °C, 5 mL per 100 g of body weight) subcutaneously. Surviving animals were euthanized on day 7–9 post-sepsis induction. MFX was dissolved in DMSO and epirubicin and meropenem were dissolved in PBS. They were aliquoted and stored at −80 °C. MFX (20 µg/g body weight), epirubicin (0.6 µg/g body weight) and meropenem (40 µg/g body weight) were injected intraperitoneally at 0 and 24 h following CLP.

### 4.6. Colony-Forming Units Assay

Blood samples from the septic and mock CLP mice were collected by cardiac puncture at indicated times after surgery. Serial dilutions of blood were immediately plated on Trypticase Soy Agar II plates supplemented with 5% Sheep Blood. CFUs were counted after 24 h of incubation at 37 °C.

### 4.7. Serology and Cytokine Measurement

Plasma from blood samples obtained 24 h post CLP was collected after centrifugation. Lactate dehydrogenase (LDH), alanine transaminase (ALT), urea and lactate levels were measured using the Biolegend^®^ KITs according to the company’s protocol. The levels of IL-1β, TNF-α and IL-6 were measured using murine R&D^®^ ELISA kits (Minneapolis, MN, USA), according to company’s protocol. 

### 4.8. Statistics

The continuous variables are expressed as means ± standard deviations (SD) and were analyzed using Student’s *t*-test. The survival proportions were analyzed using the log-rank test. *p* ≤ 0.05 was considered statistically significant.

## 5. Conclusions

MFX effectively reduces TNF-α and IL-1β levels in immune cells stimulated with LPS and *E. coli*. Moreover, it exhibits protective properties in an animal model of sepsis, resulting in a significant reduction in cytokines and markers of organ damage. These effects appear to be independent of its antimicrobial activity and are not associated with DNA damage. The molecular mechanisms underlying its immunomodulatory effects remain elusive, presenting an intriguing direction for future research studies.

## Data Availability

The data generated in this research will be shared upon reasonable request to the corresponding author.
